# Spectroscopic and Molecular Docking Studies of Cu(II), Ni(II), Co(II), and Mn(II) Complexes with Anticonvulsant Therapeutic Agent Gabapentin

**DOI:** 10.3390/molecules27134311

**Published:** 2022-07-05

**Authors:** Moamen S. Refat, Ahmed Gaber, Yusuf S. Althobaiti, Hussain Alyami, Walaa F. Alsanie, Sonam Shakya, Abdel Majid A. Adam, Mohamed I. Kobeasy, Kareem A. Asla

**Affiliations:** 1 Department of Chemistry, College of Science, Taif University, P.O. Box 11099, Taif 21944, Saudi Arabia; majidadam@tu.edu.sa (A.M.A.A.); m.kobeasy@tu.edu.sa (M.I.K.); 2 Department of Biology, College of Science, Taif University, P.O. Box 11099, Taif 21944, Saudi Arabia; 3 Centre of Biomedical Sciences Research (CBSR), Deanship of Scientific Research, Taif University, P.O. Box 11099, Taif 21944, Saudi Arabia; w.alsanie@tu.edu.sa; 4 Department of Pharmacology and Toxicology, College of Pharmacy, Taif University, P.O. Box 11099, Taif 21944, Saudi Arabia; ys.althobaiti@tu.edu.sa; 5 Addiction and Neuroscience Research Unit, Taif University, P.O. Box 11099, Taif 21944, Saudi Arabia; 6 College of Medicine, Taif University, P.O. Box 11099, Taif 21944, Saudi Arabia; hmyami@tu.edu.sa; 7 Department of Clinical Laboratories Sciences, the Faculty of Applied Medical Sciences, Taif University, P.O. Box 11099, Taif 21944, Saudi Arabia; 8 Department of Chemistry, Faculty of Science, Aligarh Muslim University, Aligarh 202002, India; sonamshakya08@gmail.com; 9 Department of Chemistry, Faculty of Science, Zagazig University, Zagazig 44519, Egypt; kareemnano2014@gmail.com

**Keywords:** gabapentin, transition metals, spectroscopic, FTIR, electronic spectra, TEM

## Abstract

New Cu(II), Ni(II), Co(II), and Mn(II) complexes of the gabapentin (Gpn) bidentate drug ligand were synthesized and studied using elemental analyses, melting temperatures, molar conductivity, UV–Vis, magnetic measurements, FTIR, and surface morphology (scanning (SEM) and transmission (TEM) electron microscopes).The gabapentin ligand was shown to form monobasic metal:ligand (1:1) stoichiometry complexes with the metal ions Cu(II), Ni(II), Co(II), and Mn(II). Molar conductance measurements in dimethyl-sulfoxide solvent with a concentration of 10^−3^ M correlated to a non-electrolytic character for all of the produced complexes. A deformed octahedral environment was proposed for all metal complexes. Through the nitrogen atom of the –NH_2_ group and the oxygen atom of the carboxylate group, the Gpn drug chelated as a bidentate ligand toward the Mn^2+^, Co^2+^, Ni^2+^, and Cu^2+^ metal ions. This coordination behavior was validated by spectroscopic, magnetic, and electronic spectra using the formulas of the [M(Gpn)(H_2_O)_3_(Cl)]·*n*H_2_O complexes (where *n* = 2–6).Transmission electron microscopy was used to examine the nanostructure of the produced gabapentin complexes. Molecular docking was utilized to investigate the comparative interaction between the Gpn drug and its four metal [Cu(II), Ni(II), Co(II), and Mn(II)] complexes as ligands using serotonin (6BQH) and dopamine (6CM4) receptors. AutoDock Vina results were further refined through molecular dynamics simulation, and molecular processes for receptor–ligand interactions were also studied. The B3LYP level of theory and LanL2DZ basis set was used for DFT (density functional theory) studies. The optimized geometries, along with the MEP map and HOMO → LUMO of the metal complexes, were studied.

## 1. Introduction

**Figure 1:**
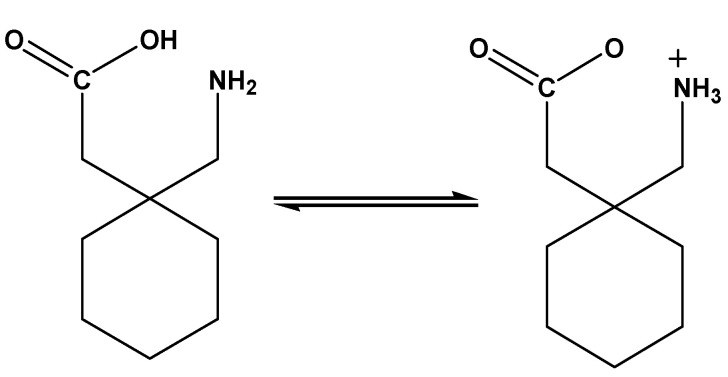
The zwitterion structure of the gabapentin (Gpn) drug.

GABAPENTIN (Gpn; Figure 1), is sold under the brand name neurontin, and is commonly named as 2-[1-(aminomethyl)cyclohexyl]acetic acid. Gpn is a structural analogue of the neurotransmitter gamma aminobutyric acid, which has mostly been investigated for its inhibitory effect on the central nervous system [1]. Since Gpn has both acidic groups (COOH) and a basic group (NH_2_), it is an artificial amino acid [1,2]. It is a antiepileptic drug that is utilized as both a supplement and a stand-alone treatment for people suffering from partial seizures [2]. Gpn has also been used in the treatment of neuropathic pain. For a growing number of individuals with epilepsy, a safe and effective seizure is a major concern. Epilepsy has a significant economic impact [3]. The effectiveness, tolerability, and safety of an antiepileptic medicine are all factors to consider when choosing one. Gpn can also be regarded as an emergent solution to the “pain puzzle”. Double-blind and more randomized studies comparing analgesic medications to Gpn may be useful in determining the first-line treatment for chronic and acute pain relief [4]. As a result, finding compounds to treat epilepsy is necessary [5,6]. Metal-binding or metal-recognition sites are found in a variety of drugs and potential pharmacological agents. These sites can bind or interact with metal ions, influencing their bioactivities and possibly causing damage to their target biomolecules. The literature [7,8,9,10] has numerous examples of these “metallodrugs” and “metallopharmaceuticals” and their actions. Metals and metal complexes have played an important part in the evolution of contemporary chemotherapy. Anticancer platinum drugs, for example, are used in more chemotherapy regimens than any other class of anticancer drugs, and they have played a significant role in the success of cancer treatment over the last three decades [11].

After coordinating to a metal, *ligands* can play an essential role in changing the pharmacological characteristics of existing medications. Because the resulting prodrugs have varied physical and pharmacological properties, they can be delivered in a regulated manner or at a specific site [7]. This method can be used to save medications that have failed due to poor pharmacology or high toxicity. The complexation of nonsteroidal anti-inflammatory medications to copper, for example, eliminates some of the drugs’ gastrointestinal adverse effects. In the hypoxic zones of solid tumors, the release of cytotoxins such as nitrogen mustards from redox-active metals such as cobalt has the potential to improve medication effectiveness and reduce toxicity [8].

Metal complexes have received less attention in medical chemistry than organic molecules [9]. Many organic chemicals employed in medicine, in reality, do not have a wholly organic mechanism of action and require the residues of metal ions for activation or biotransformation, either directly or indirectly. The status of metal ions and their complexes with biomolecules in the body affects our health, aging, physiological problems, and diseases. Metals make up about 0.03% of the weight of the human body. The concentrations of Cd, Cr, Ti, V, Cu, Se, and Zn in malignant sections of the kidney were shown to be lower than in noncancerous parts [10]. Coordination bonds can be formed by ligands with electron donor atoms such as N, O, S, and P. Chelation alters the biological properties of both ligands and metal moieties, and in many circumstances, it produces a synergistic interaction between the metal ion and the ligand [10].

Mechanosynthesis has been used to create a variety of Gpn coordination networks with Mn^2+^, Y^3+^, La^3+^, Er^3+^, Nd^3+^, and Ce^3+^ [11,12]. In the literature survey, there appears to be a lack of study on the chelation behavior of gabapentin and its derivatives toward metal ions [13,14]. Therefore, in this paper, the experimental plan aimed to synthesize some new Cu(II), Ni(II), Co(II), and Mn(II) complexes of gabapentin by refluxing some transition metal(II) chlorides with a basic solution of gabapentin in a 1:1 molar ratio in a methanol solvent. The structure of the obtained complexes was studied using spectral, magnetic, and morphological techniques. AutoDock Vina software was used to study the interactions between the receptors (serotonin and dopamine) and ligands [gabapentin (Gpn) and its four synthesized metal complexes ([Cu(II)–(Gpn)], [Ni(II)–(Gpn)], [Co(II)–(Gpn)], and [Mn(II)–(Gpn)]) were studied theoretically by the molecular docking method. For more clarity, the binding energy along with the aromatic, hydrogen bond, hydrophobic, SAS, ionizability, and interpolated charge surfaces at the interaction site were also generated. The molecular docking outputs were further investigated for receptor–ligand interactions through MDS (molecular dynamic simulation), which used a 100 ns run at 300 K. Structural stability, hydrogen bond interactions, SASA, compactness of structure, and residue flexibility of the complexes were studied to compare their dynamic features. The optimized geometries of ([Cu(II)–(Gpn)], [Ni(II)–(Gpn)], [Co(II)–(Gpn)], and [Mn(II)–zx(Gpn)] were obtained through the B3LYP level of theory and LanL2DZ basis set of the DFT calculations. Important parameters such as chemical, structural, and spectroscopic properties of the [Cu(II)–(Gpn)] complex were also obtained.

## 2. Materials and Methods

### 2.1. Chemicals

Metal chloride salts (MnCl_2_.4H_2_O (≥99%), CoCl_2_.6H_2_O (98%), CuCl_2_.2H_2_O (≥99%), and NiCl_2_.6H_2_O (≥98%)) and gabapentin (≥99%) were obtained from Sigma-Aldrich, USA and utilized in the preparation as received.

### 2.2. Instruments

The following is a list of the different types of analysis and their respective models:
**Type of Analysis****Models**SEMQuanta FEG 250 equipmentTEMJEOL 100s microscopyMagnetic momentMagnetic Susceptibility BalanceElectronic spectraUV2 Unicam UV/Vis SpectrophotometerFTIR spectraBruker FTIR SpectrophotometerConductanceJenway 4010 conductivity meterElemental analyses (C,H,N)Perkin Elmer CHN 2400Metal ions (Mn, Co, Cu, Ni)An atomic absorption spectrometer model PYE-UNICAM SP 1900


### 2.3. Synthesis of Gpn Metal Complexes

#### 2.3.1. Synthesis of the [Mn(Gpn)(H_2_O)_3_(Cl)]·4H_2_O Complex

The synthesis of the manganese(II)complex was carried out by adding MnCl_2_·4H_2_O (1.98 g, 1.0 mmol) to the basic solution of the gabapentin ligand (Gpn) (1.72 g, 1.0 mmol) by neutralization (pH = ~7.0) using NH_3_ solution. The mixture was stirred at 80 °C for 3 h. The dark brown precipitate of the manganese(II) complex was filtered and washed with distilled water three times, dried, and kept in desiccators. Yield: 77%. C_9_H_30_ClMnNO_9_, Mwt. 386.73 g/mol (Found: C 27.87; H 7.76; N 3.59; Mn 14.08; Mwt. 386.73, calcd. C 27.95; H 7.82; N 3.62; Mn 14.21; m.p. > 234 °C).

#### 2.3.2. Synthesis of [Co(Gpn)(H_2_O)_3_(Cl)]·6H_2_O Complex

The synthesis of the cobalt(II) complex was carried out by adding CoCl_2_·6H_2_O (2.38 g, 1.0 mmol) to the basic solution of the gabapentin ligand (Gpn) (1.72 g, 1.0 mmol) by neutralization (pH = ~7.0) using the NH_3_ solution. The mixture was stirred at 80 °C for 2 h. The dark blue precipitate of the cobalt(II) complex was filtered and washed with distilled water three times, dried, and kept in desiccators. Yield: 70%. C_9_H_34_ClCoNO_11_, Mwt. 426.75 g/mol (Found: C 25.23; H 7.98; N 3.24; Co13.76; Mwt. 426.75, calcd. C 25.33; H 8.03; N 3.28; Co13.81; m.p. > 237 °C).

#### 2.3.3. Synthesis of [Cu(Gpn)(H_2_O)_3_(Cl)]·2H_2_O Complex

The synthesis of copper(II) complex was carried out by adding CuCl_2_·2H_2_O (1.71 g, 1.0 mmol) to the basic solution of gabapentin ligand (Gpn) (1.72 g, 1.0 mmol) by neutralization (pH = ~7.0) using the NH_3_ solution. The mixture was stirred at 80 °C for 2 h. The dark brown precipitate of the copper(II) complex was filtered and washed with distilled water three times, dried, and kept in desiccators. Yield: 72%. C_9_H_26_ClCuNO_7_, Mwt. 359.30 g/mol (Found: C 30.03; H 7.16; N 3.87; Cu17.54; Mwt. 359.30, calcd. C 30.08; H 7.29; N 3.90; Cu17.69; m.p. > 232 °C).

#### 2.3.4. Synthesis of [Ni(Gpn)(H_2_O)_3_(Cl)]·3H_2_O Complex

The synthesis of the nickel(II) complex was carried out by adding NiCl_2_·6H_2_O (2.38 g, 1.0 mmol) to the basic solution of the gabapentin ligand (Gpn) (1.72 g, 1.0 mmol) by neutralization (pH = ~7.0) using the NH_3_ solution. The mixture was stirred at 80 °C for 2 h. The green precipitate of the nickel(II) complex was filtered and washed with distilled water three times, dried, and kept in desiccators. Yield: 75%. C_9_H_28_ClNNiO_8_, Mwt. 372.47 (Found: C 29.00; H 7.46; N 3.71; Ni 17.65; Mwt. 372.47, calcd. C 29.02; H 7.58; N 3.76; Ni 15.76; m.p. > 238 °C).

### 2.4. Computational Studies

#### 2.4.1. Molecular Docking

All of the docking was conducted through an Intel(R) Core(TM) i5-4200U CPU-2.10 GHz, 64 bit processor. AutoDock Vina [15] program was used to perform the docking. The receptors serotonin and dopamine with PDB ID: 6BQH and 6CM4, respectively, were obtained through an online PDB bank [16]. Serotonin and dopamine were set up by cleaning the ligand and water molecules through Discovery Studio (DS) software (https://www.3ds.com/products-services/biovia/, accessed on 1 Febrauary 2022). DTool [17] was used to add polar hydrogen atoms and Kollman charges, while the Geistenger method was used to assign the partial charges. The ligands [gabapentin (Gpn) and its four complexes—([Mn(II)–(Gpn)], [Co(II)–(Gpn)], [Ni(II)–(Gpn)], and [Cu(II)–(Gpn)])]—in the PDBQT format were obtained using the OpenBabelIGUI program [18]. The energy of all of the ligands was minimized through PyRx-Python at 200 steps using the conjugate gradient optimization algorithm and MMFF94 force field [19]. Discovery Studio software was used to analyze the docking results.

#### 2.4.2. Molecular Dynamics Simulation (MDS) Studies

To conduct the simulation, an “Intel(R) Xeon(R) CPU E5-2680 v4-2.40GHz, 64 bit” processor was used. MDS was performed using GROMACS version 2019.2 with a GROMOS96-43a1 force field. To initiate the MDS analysis, results obtained from molecular docking for Gpn and [Cu(II)–(Gpn)] with a high docking score were used. Topology and parameter files were created through CHARMM-GUI with the latest CGenFF [20,21]. To solve receptor–ligand structures in a triclinic box, SPC water models were used [22]. Twenty-seven Cl^-^ and 28 Na^+^ ions were added for neutralization at 0.15 M salt (Figure S1). A Leap-frog MD integrator for a 100 ns simulation time in the NPT/NVT equilibration run systems was exposed to the periodic boundary conditions (300 K and 1.0 bar) [23,24]. The RMSD (root mean square deviation), hydrogen bonding, SASA, and gyration radius were examined using gmx rms, gmxhbond, gmxsasa, and gmx gyrate tools, respectively [25]. PyMol/VMD and Grace Software [12,13] were used to prepare the different plots.

#### 2.4.3. Density Functional Theory (DFT)

The Gaussian 09RevD.01 package [26] was used for the DFT/TD-DFT calculations. The molecules were optimized in their ground spin state B3LYP density functional to know the exact position of the hydrogen atoms, as B3LYP, a hybrid method containing elements from DFT and the Hartree–Fock theory, was the first DFT exchange correlation functional to convince computational chemists that DFT could predict molecule physicochemical properties and reaction barriers with an accuracy comparable to some wave function-based methods but with much improved computational efficiency. The Los Alamos Effective Core Potentials lanL2DZ basis set was employed for the Mn, Co, Ni, and Cu atom while the split-valence 6-31G(d) basis set was applied for the other atoms [27]. A molecular electrostatic potential map (MEP) of the synthesized metal complexes were studied [28]. The IR frequencies were computed with full accuracy using animated modes of vibrations. To establish a good comparison between the experimental and theoretical wavenumbers, the scaling factor of 0.8522 was used. Moreover, structure-based molecular properties such as bond lengths, bond angles, atomic charges, total energy, electronic properties, and frontier molecular orbitals energy were calculated by this theory in the gas phase. ChemCraft 1.5 software [29] was used for visualization.

## 3. Results and Discussion

Herein, our paper aimed to synthesize a complexation between the gabapentin pure drug and four transition metal ions with a 1:1 molar ratio in alkaline media and check the suggested structures of the resulting complexes by spectroscopic characterizations. The difference between our synthesized complexes and others mentioned in the literature is the isolated 1:1 molar ratio complexation and the isolation at pH = 7. The articles mentioned in the literature dealing with the mixed gabapentin ligands and the isolation of the 1:2 molar ratio were different from our synthesized complexes. We looked at a lot of crystallites but could not find one that was adequate for full data capture. As a result, a complete dataset of nickel(II), cobalt(II), copper(II), and manganese(II) gabapentin complexes was not collected, and the discussion was based on the elemental analysis, melting points, molar conductivity, UV–Vis, magnetic measurements, FTIR, and surface morphology (scanning (SEM) and transmission (TEM) electron microscopes) as well as the theoretical study.

### 3.1. Elemental and Conductance Measurements

**Figure 2:**
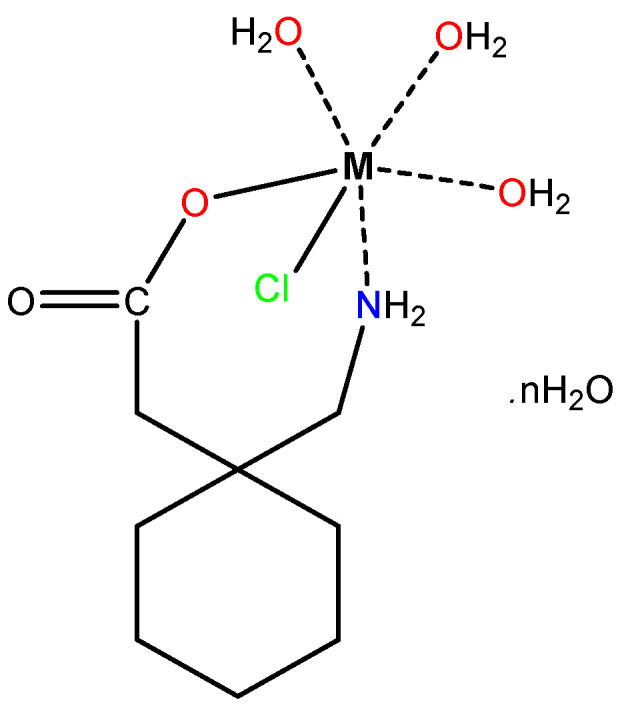
The structures of the synthesized Mn(II), Co(II), Ni(II), and Cu(II)–Gpn complexes, where = 2 for Cu(II), 3 for Ni(II), 4 for Mn(II), and 6 for Co(II).

The gabapentin metal complexes of nickel(II), cobalt(II) copper(II), and manganese(II) are commonly soluble in DMSO (dimethyl sulfoxide) and DMF (dimethylformamide). The synthesized complexes had a stoichiometry ratio of 1:1 (metal:ligand), according to the elemental analytical data; the postulated structure of the complexes is well-supported by analytical data and other spectrum studies (Figure 2). The color, yield, and melting point data of all the compounds were introduced in experimental Section 2.3. In DMSO, the molar conductance of the solutions of all the complexes were in the range of 11–14 Ω^−1^cm^2^ mole^−1^. These experimental data indicate that complexes are non-electrolytes [30] in DMSO (10^−3^ M) at room temperature. Metal ions and Gpn ligand reactions with a 1:1 molar ratio yielded bidentate complexes. The Gpn ligand on reaction with Cu(II), Ni(II), Co(II), and Mn(II) salt yielded complexes that corresponded to the formulas [Cu(Gpn)(H_2_O)_3_(Cl)]·2H_2_O, [Ni(Gpn)(H_2_O)_3_(Cl)]·3H_2_O, [Co(Gpn)(H_2_O)_3_(Cl)]·6H_2_O, and [Mn(Gpn)(H_2_O)_3_(Cl)]·4H_2_O. The general compositions for the complexes are [M(Gpn)(H_2_O)_3_(Cl)]·nH_2_O complexes (where *n* = 2–6). Elemental analyses of the prepared compounds indicate the formation of six coordination complexes containing one molecule of gabapentin as a bidentate ligand, one chlorine atom, and three water molecules. The absence of an absorption band at ca. 3400 cm^−1^ in the IR spectrum of the Gpn ligand shows that free amino groups were absent. A broad band was observed at ca. 1650–1680 cm^−1^, which indicates the presence of water molecules from the complexes.

**Figure 3:**
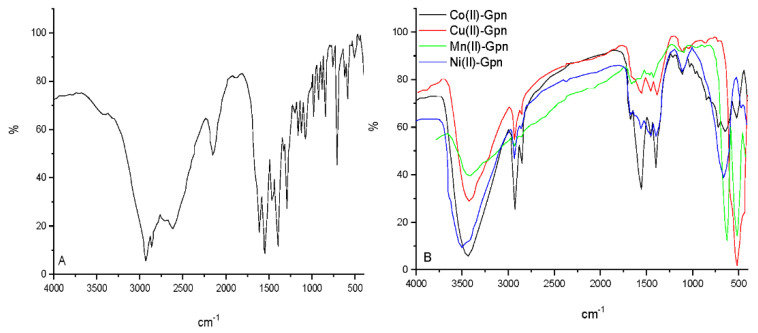
The infrared spectra of (**A**) Gpn free drug and (**B**) Mn(II), Co(II), Ni(II), and Cu(II)–Gpn complexes.

The FTIR spectra of the pure gabapentin and each of the Cu(II), Ni(II), Co(II), and Mn(II)–Gpn complexes were also obtained (Figure 3 and Table 1).

Regarding the infrared spectrum of the free gabapentin ligand, it did not show a peak of the stretching –NH within the region (3500–3300 cm^−1^), since it is a zwitterion in the solid state [16,17]. The two main characteristic bands at 2928 and 2861 cm^−1^ were assigned to the –NH_3_^+^ stretching vibrations [31,32]. The peak at 2150 cm^−1^ was for the CN group side chain stretching vibration [32]. At 1614 and 1545 cm^−1^, the peaks represented the stretching asymmetric vibration and vibrations of the NH_3_^+^ bending deformation of the carboxylate group, respectively [32]. In the 400–4000 cm^−1^ region, the FTIR spectra of the gabapentin complexes exhibited several bands. The δ(NH_2_) bond vibration appeared at 1552–1568 cm^−1^ for the copper complex, which showed a shift to lower frequencies to free Gpn [33]. To identify the type of carboxylate binding to a transition metal ion, the value Δν = [ν_as_(COO) − ν_s_(COO)] was employed. Generally, unidentate carboxylation is indicated by the difference in Δν between the symmetric and asymmetric (COO) absorption frequencies of >200 cm^−1^. In the nickel(II), cobalt(II), copper(II), and manganese(II) complexes, the Δν values were 283, 274, 270, and 240, cm^−1^, indicating monodendate coordination of the carboxylate group [34]. In these synthesized complexes, it can be attributed to the Gpn ligand, which is linked to the metal ion by carboxylic acid atoms and the nitrogen of amino groups. Furthermore, the existence of ν(M–O) and ν(M–N) medium intensity bands at 612–620 cm^−1^ and 468–519, respectively, suggested the generation of Gpn complexes [35]. In the FTIR spectra, the characteristic bands of bending to water hydrated, δ(H_2_O) at ca. 1650 cm^−1^ and broad absorption bands of (OH) with maximum at 3404–3507 cm^−1^ in synthesized metal complexes confirmed the presence of coordinated water molecules.

### 3.2. Electronic Spectra, and Magnetic Susceptibility Studies

#### 3.2.1. Manganese(II) Complex

The molar conductance measurements showed that the Mn(II) complex is a non-electrolyte. Thus, it is formulated as [Mn(Gpn)(H_2_O)_3_(Cl)] 4H_2_O. It showed a magnetic moment corresponding to five unpaired electrons (5.87 B.M.) at room temperature, which is close to the spin only value (5.92 B.M.). The electronic spectrum of this complex displayed weak absorption bands at 568 nm (ν1), 388 nm (ν2), 378 nm (ν3), and 369 nm (ν4), characteristic of octahedral geometry [36]. These bands may be assigned as ^6^A_1g_ → ^4^T_1g_, ^6^A_1g_ → ^4^E_g_, ^4^A_1g_, ^6^A_1g_ → ^4^E_g_ and ^6^A_1g_ → ^4^T_1g_ transitions, respectively.

#### 3.2.2. Cobalt(II) Complex

The molar conductance measurements showed that the complex is also a non-electrolyte. Thus, it may be formulated as [Co(Gpn)(H_2_O)_3_(Cl)] 6H_2_O. At room temperature, it showed a magnetic moment of 4.90 B.M., a value in tune with a high-spin configuration showing the presence of a distorted octahedral environment around the cobalt(II) ion in the complex. The electronic spectrum of the complex displayed absorption bands at 610 and 542 nm. A distorted octahedral geometry, this complex is indicated through the investigation of this electronic spectral data [37]. The assignment of the spectral bands may be given as ^4^T_1_(F) → ^4^A_2_(F) and ^4^T_1_(F) → ^4^T_1_(P) transitions, respectively.

#### 3.2.3. Copper(II) Complex

The molar conductance measurements showed that the complex is a non-electrolyte and that it may be formulated as [Cu(Gpn)(H_2_O)_3_(Cl)] 2H_2_O. The magnetic moment of the complex was 1.96 B.M. The six-coordinate copper(II) complex had either D_4h_ or C_4v_ symmetry, and the E_g_ and T_2g_ levels of the 2D free ion split into B_1g_, A_1g_, B_2g_, and E_g_ levels, respectively. Thus, three spin-allowed transitions are expected in the visible and near-IR region, but only a few complexes are known in which such bands are resolved [38]. These bands were assigned to the following transitions, in order of increasing energy, ^2^B_1g_ → ^2^A_1g_, ^2^B_1g_ → ^2^B_2g_, and ^2^B_1g_ → ^2^E_g_. The energy level sequence will depend on the amount of tetragonal distortion due to ligand-field and Jahn–Teller effects [39]. The electronic spectrum of the complex showed a band at 722 nm and a well-defined shoulder at 620 nm, which may be assigned to the ^2^B_1g_ → ^2^A_1g_ and ^2^B_1g_ → ^2^E_g_ transitions, respectively.

#### 3.2.4. Nickel(II) Complex

Nickel(II) has a (d^8^) configuration with the following orgel diagram found at 780 nm, 398 nm, and 384 nm, respectively, for the nickel complex. Based on the orgel diagram, the first peak at 697 nm was assigned to ^3^A_2g_ → ^3^T_2g_, the second at 346 nm for ^3^A_2g_ → ^3^T_1g_ (F), and the third at 315 nm was due to the ^3^A_2g_→^3^T_1g_ (P) transition, respectively. This confirms the presence of an octahedral geometry for the nickel complex. The magnetic moment of the complex was 3.28 B.M. at room temperature, a value in tune with a high-spin configuration, showing the presence of a distorted octahedral environment around the nickel(II) ion in the complex. On the basis of magnetic, molar conductivity, molar ratio, UV/Vis., and the IR results, the proposed molecular structure of the synthesized complexes is octahedral (Figure 3) [37].

### 3.3. Scanning and Transmission Electron Microscopes

**Figure 4:**
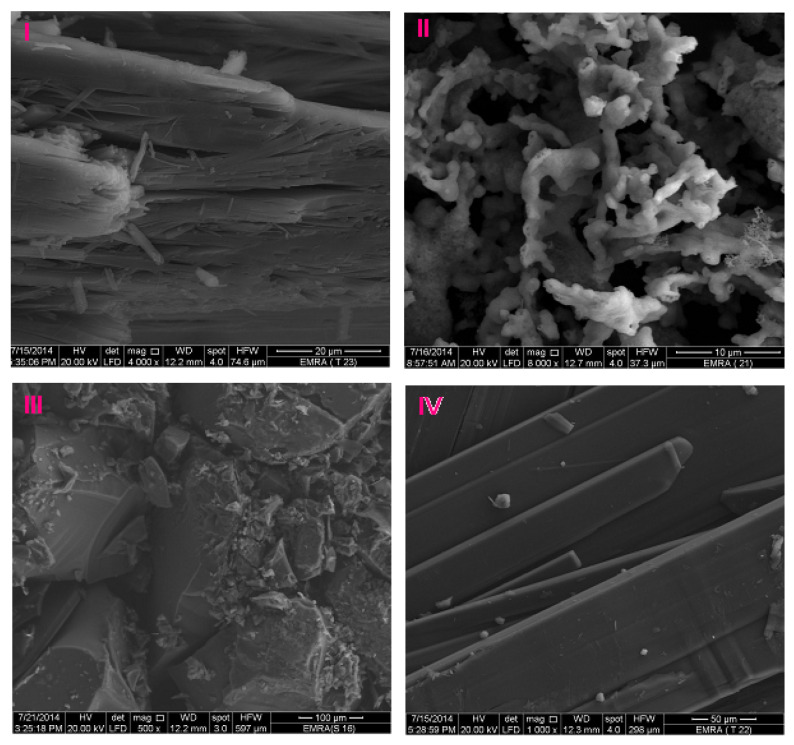
The SEM images of (**I**) Mn(II), (**II**) Cu(II), (**III**) Co(II), and (**IV**) Ni(II)–Gpn.

**Figure 5:**
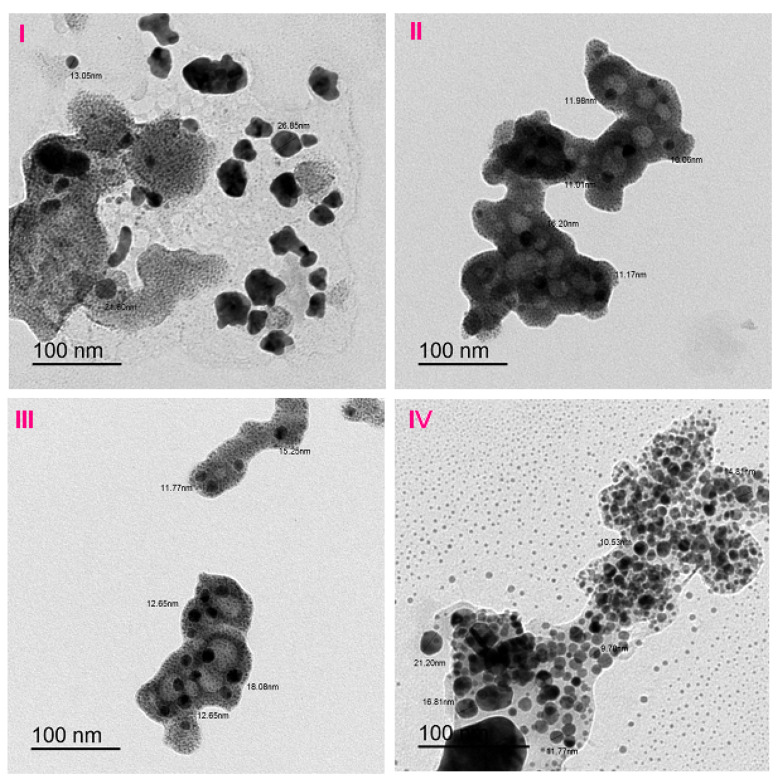
The TEM images of the (**I**) Mn(II), (**II**) Cu(II), (**III**) Co(II), and (**IV**) Ni(II)–Gpn complexes.

The SEM analysis was carried out to check the surface morphology of the selected complexes, and the micrographs obtained are given in Figure 4. The micrographs of the manganese(II), copper(II), cobalt(II), and nickel(II)–gabapentin complexes are given in Figure 5I–IV; it can be seen that these gave an appearance of pressed chips (a wooden board has several layers), a coral reef-like, a block of rock, and rectangular panels of different sizes, respectively.

The TEM image (Figure 5I–IV) shows that the Mn(II)–Gpn, Cu(II)–Gpn, Co(II)–Gpn, and Ni(II)–Gpn complexes were aggregates with an irregular shape. The particle sizes of the [Mn(Gpn)(H_2_O)_3_(Cl)] 4H_2_O, [Co(Gpn)(H_2_O)_3_(Cl)]·6H_2_O, [Cu(Gpn)(H_2_O)_3_(Cl)] 2H_2_O, and [Ni(Gpn)(H_2_O)_3_(Cl)] 3H_2_O complexes were calculated from the TEM images with the ranges of 13–27 nm, 12–18 nm, 10–18 nm, and 10–20 nm, respectively (Figure 5).

### 3.4. Molecular Docking Studies

The interactions between the synthesized metal complexes—([Cu(II)–(Gpn), [Ni(II)–(Gpn)], [Co(II)–(Gpn)], and [Mn(II)–(Gpn)]]—with the prepared serotonin and dopamine receptors were studied, and the best docking poses were analyzed. For comparative purposes, the Gpn drug was used as the control. The results revealed that the potential binding energy of all of the metal complexes was higher than Gpn in both the receptors (Table 2).

**Figure 6:**
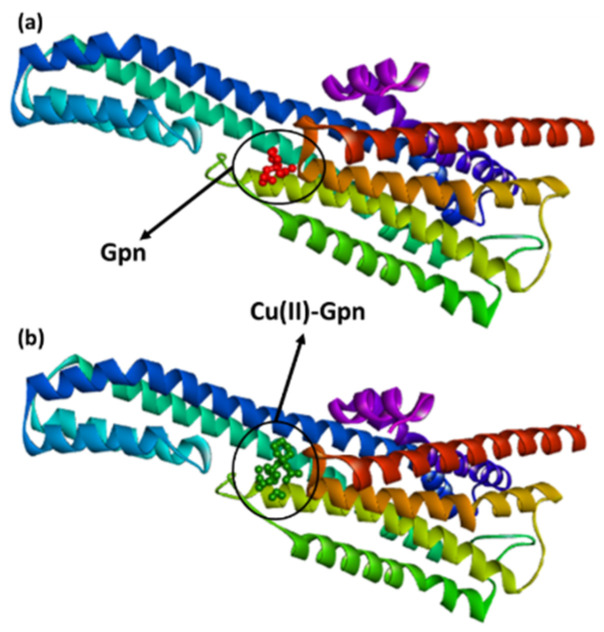
The best docked pose showing a helical model of serotonin (PDB ID: 6BQH) docked with (**a**) the Gpn drug and (**b**) metal complex [Cu(II)–(Gpn)].

Among the four metal complexes screened, [Cu(II)–(Gpn)] showed the highest docking energy. Docking of [Cu(II)–(Gpn)] with serotonin and dopamine gave the potential binding energy of −7.2 and −6.5 kcal/mol, respectively. The greater value of the binding energy in the case of [Cu(II)–(Gpn)]-serotonin (**CuGS**) suggests a stronger interaction than with dopamine. The docking data are given in Table 3, and the best docking pose (**CuGS**) is shown in Figure 6.

**Figure 7:**
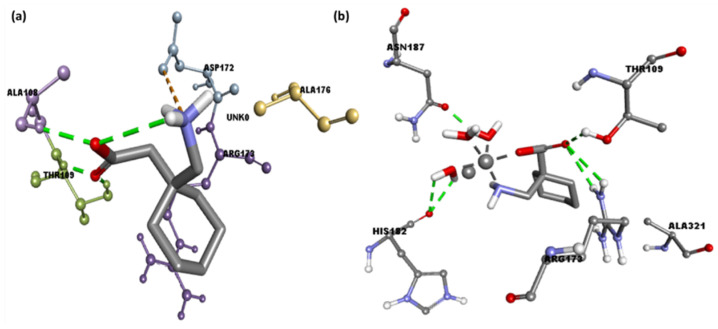
The 3D representation of the interactions for serotonin (PDB ID: 6BQH) docked with (**a**) the Gpn drug and (**b**) metal complex [Cu(II)–(Gpn)].

An illustration of the molecular docking for the interactions of the ligands and receptor is depicted in Figure 7a,b.

**Figure 8:**
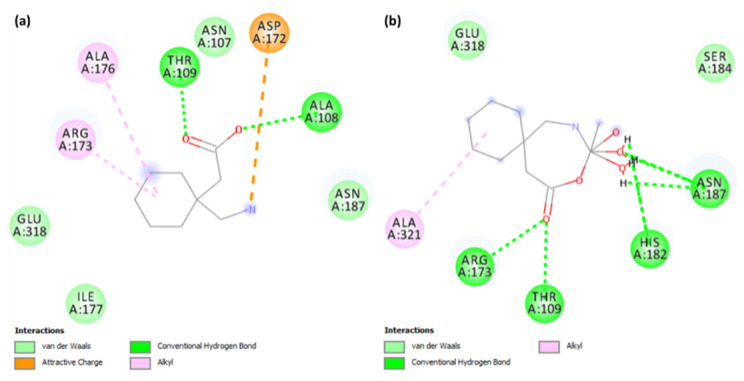
The 2D representation of interactions for serotonin (PDB ID: 6BQH) docked with (**a**) the Gpn drug and (**b**) metal complex [Cu(II)–(Gpn)].

As shown in Figure 8a, **CuGS** showed that the residues Arg173, Thr109, His182, and Asn187 had established hydrogen bonds. Additionally, Asp172 (Attractive charge) and Ala176 and Arg173 (Alkyl) interactions could also be seen [40,41]. Similarly, docking of the Gpn drug with serotonin and dopamine gave the potential binding energy of −5.1 and −4.8 kcal/mol, respectively. The greater value of the binding energy in the case of Gpn-serotonin (**GpnS**) suggests a stronger interaction than that with dopamine. Figure 8b shows the interaction between Gpn and serotonin, revealing that the residues Thr109 and Ala108 had established hydrogen bonds. Additionally, Ala321 showed alkyl interactions. These findings suggest that the metal [Cu(II)–(Gpn)] binds with the receptors more efficiently than the Gpn drug. The 2D representations of the interactions between the metal complex/drug and receptor are shown in Figure 8. The other binding details of the interactions are tabularized in Table 4 and Table 5.

### 3.5. Solvent Accessible Surface, Aromatic, Ionizability, Hydrophobicity, and Hydrogen Bond Surfaces

**Figure 9:**
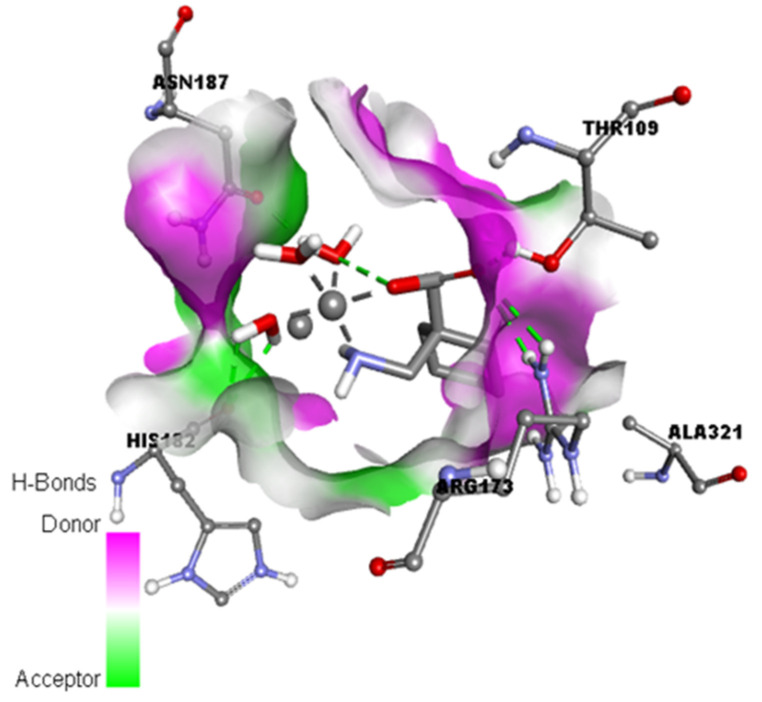
Representation of the hydrogen binding surface between the serotonin and metal complex [Cu(II)–(Gpn)].

Aromatic, hydrogen bond, hydrophobic, solvent accessible surface (SAS), ionizability, and interpolated charge surfaces at the interaction site (Figure 9 and Figure S2) were studied through DS software [42]. The aromatic shown in Figure S2a shows the face surface as an orange color and the edge surface as a blue color. The green color in the hydrogen bond surface represents the acceptor area and the pink color is the donor area of the amino acid residues (Figure 9). The presence of hydrophilicity features of the receptor around the ligand was confirmed by the hydrophobicity surface (Figure S2b). The solvent accessibility surface (SAS) is the surface area of the receptor (Figure S2c) that is reachable to a solvent [43], the green color is the poorly accessible area, and the blue color is the highly accessible area [43]. The ionization surface reflects the acidic and basic propensity (Figure S2d, blue color = basic and red color = acidic).

### 3.6. MDS Analysis

**Figure 10:**
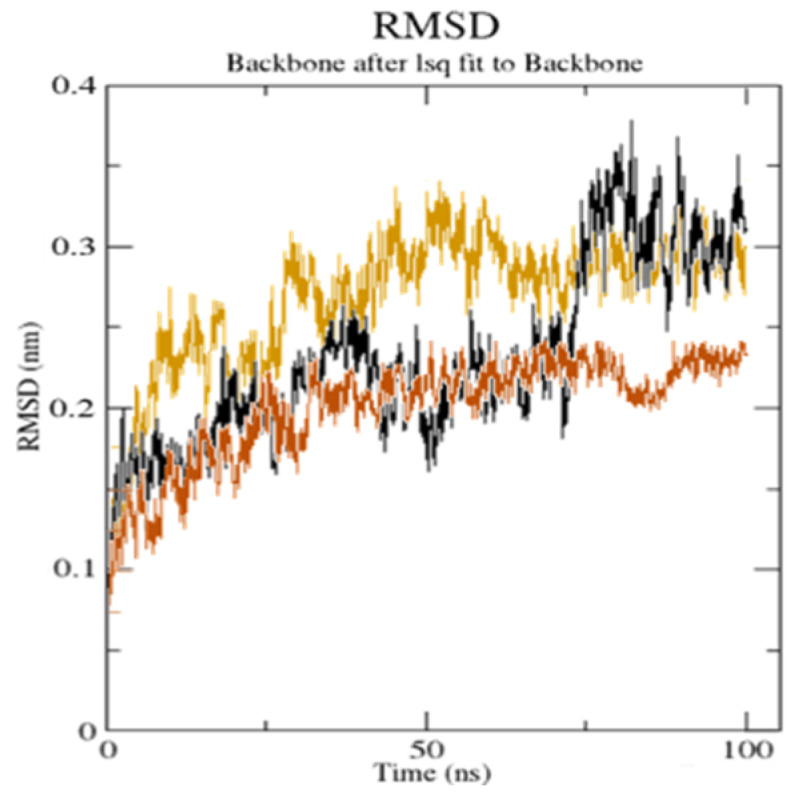
The root mean square deviation (RMSD) of the solvated receptor backbone and ligand complex during the 100 ns MD simulation [unbound serotonin receptor (yellow), **GpnS** complex (black), and **CuGS** complex (brown)]. According to the literature, a RMSD value under <3.0 Å is the most acceptable [44]. The drop in the RMSD value for **CuGS** showed an alteration in the secondary structure conformation of the protein due to the [Cu(II)–(Gpn)] interaction. This finding shows that **CuGS** developed a more stable combination. The results confirm that this interaction brings protein chains closer and reduces the gap between them, as shown in Figure 11 [45].

The top docking results (**CuGS** and **GpnS**) were used for the MDS analysis of the 100 ns run. After studying the RMSD, it was observed that both **CuGS** and **GpnS** established a stable conformation after ~30 and ~75 ns, respectively, having appropriate values of RMSD of 2.15 and 2.75 Å, respectively (Figure 10).

**Figure 12:**
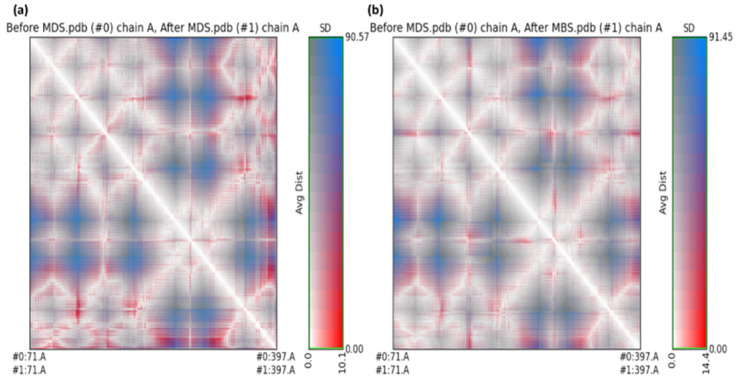
The RR distance map displaying patterns of spatial interactions between (**a**) the unbound serotonin receptor and the serotonin receptor after the simulation for **GpnS**; and (**b**) the unbound serotonin receptor and serotonin receptor after simulation for **CuGS**, showing the average distance and standard deviation for all amino acid pairs. (MDS = Molecular Dynamics simulation).

The average distance and standard deviation for all amino acid pairs between the two conformations were investigated using RR distance maps, which are two-dimensional representations of a protein’s 3D structure [46]. Figure 12 represents the RR distance maps, which plot patterns of spatial interactions [47,48].

**Figure 13:**
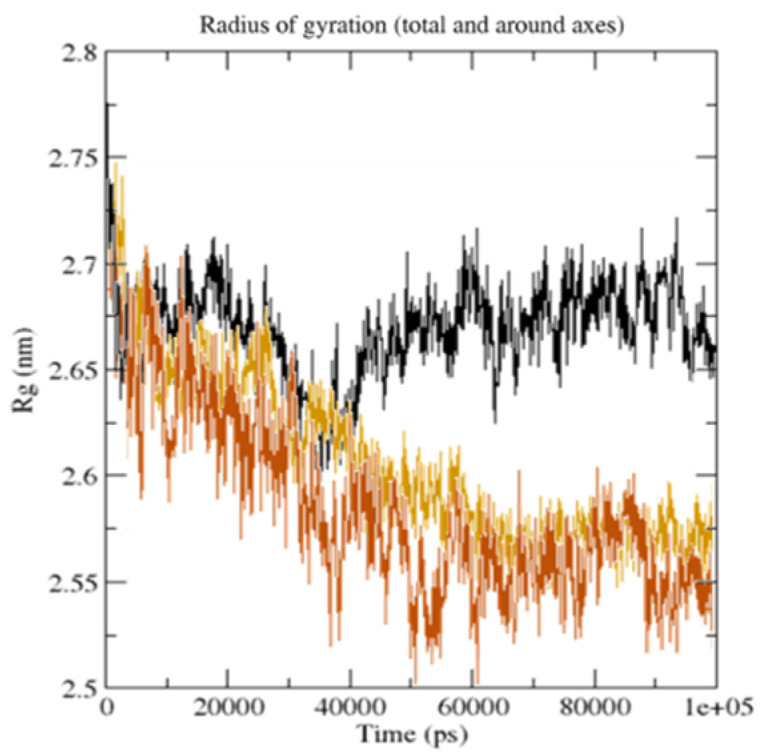
The radius of gyration (Rg) for the unbound serotonin receptor (yellow), **GpnS** complex (black), and **CuGS** complex (brown) during the 100 ns simulation time.

The zero distance between two residues is shown by the white diagonal on the map, whereas the red and blue components reflect residue pairs with the highest distance differences in the two conformations. The average radius of gyration (Rg) value of 25.75 and 26.75 Å was observed for **GpnS** and **CuGS**, respectively. It can be seen that over the simulation time, the Rg for **CuGS** decreased, indicating that the structures became more compact (Figure 13).

**Figure 14:**
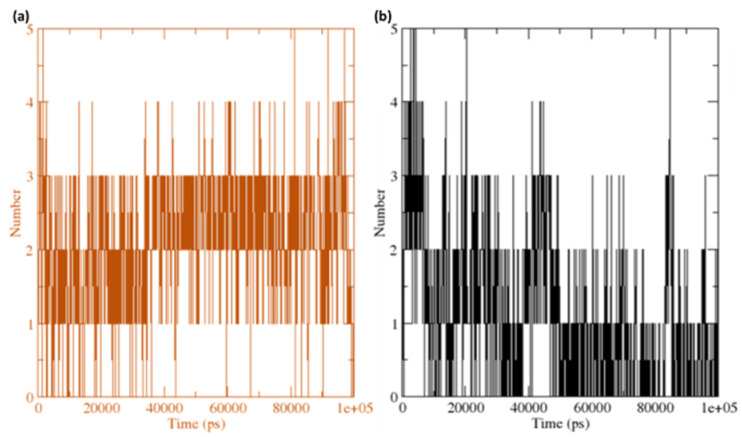
The number of average hydrogen bonding interactions between (**a**) the **CuGS** complex and (**b**) **GpnS** complex during the 100 ns simulation time.

**Figure 15:**
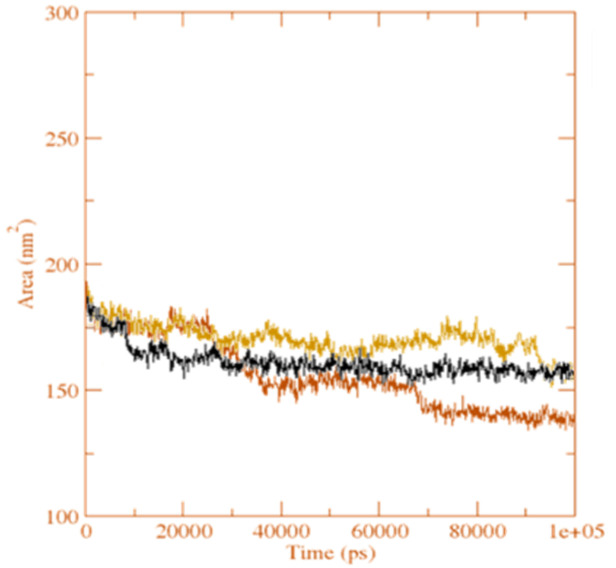
The solvent accessible surface area analysis for the unbound serotonin receptor (yellow), **GpnS** complex (black), and **CuGS** complex (brown) during the 100 ns simulation time.

The grid search at 25 × 11 × 14 = grid and rcut = 0.35 revealed the H-bond between the receptor and ligand (**GpnS** and **CuGS**), plotted against time (Figure 14). On calculating the hydrogen bonds between the ligand (Gpn and [Cu(II)–(Gpn)]) and protein (3706 atoms), 508 donors and 987 acceptors were observed. The number of H-bonds per time frame on average were found to be 2.107 and 1.104 out of a possible 250,698 for **CuGS** and **GpnS**, respectively. Overall, it was discovered that the protein–receptor interaction increased the number of hydrogen bonds substantially, which was more in **CuGS**. Figure 15 shows that the SASA (solvent accessibility surface area) values changed due to the binding of the ligand (Gpn and [Cu(II)–(Gpn)]) to the receptor (serotonin). The decreased SASA value indicates the reduced pocket size and alteration in the protein structure conformation with increased hydrophobicity.

### 3.7. DFT Calculations

**Figure 16:**
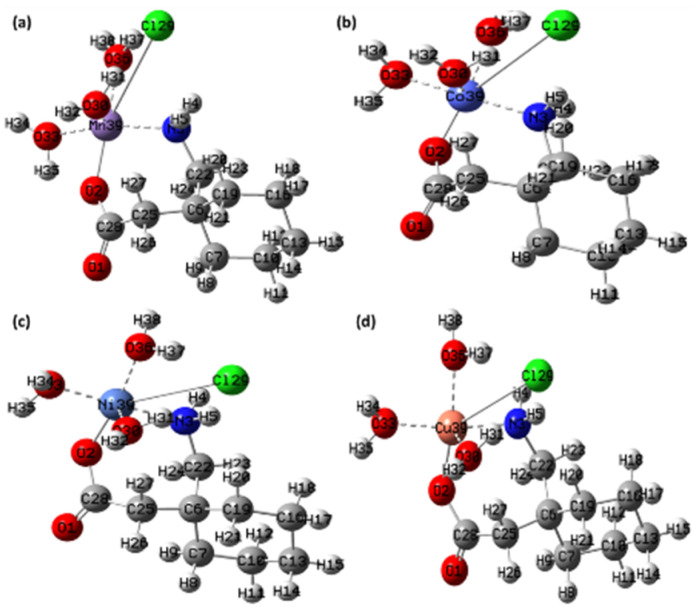
The optimized structure of the synthesized metal complexes (**a**) [Mn(II)–(Gpn)], (**b**) [Co(II)–(Gpn)], (**c**) [Ni(II)–(Gpn)], and (**d**) [Cu(II)–(Gpn)] with the Mulliken atom numbering scheme.

The structures of all four synthesized metal complexes ([Mn(II)–(Gpn)], [Co(II)–(Gpn)], [Ni(II)–(Gpn)], and [Cu(II)–(Gpn)]) were optimized using the B3LYP level of theory and LanL2DZ basis set. The minimum SCF energy after 33, 22, 22, and 40 optimization steps was found to be −906.0136, −937.0486, −961.2607, and −988.0661 a.u., respectively, for ([Mn(II)–(Gpn)], [Co(II)–(Gpn)], [Ni(II)–(Gpn)], and [Cu(II)–(Gpn)], respectively. The optimized geometry of the four metal complexes is given in Figure 16.

**Figure 17:**
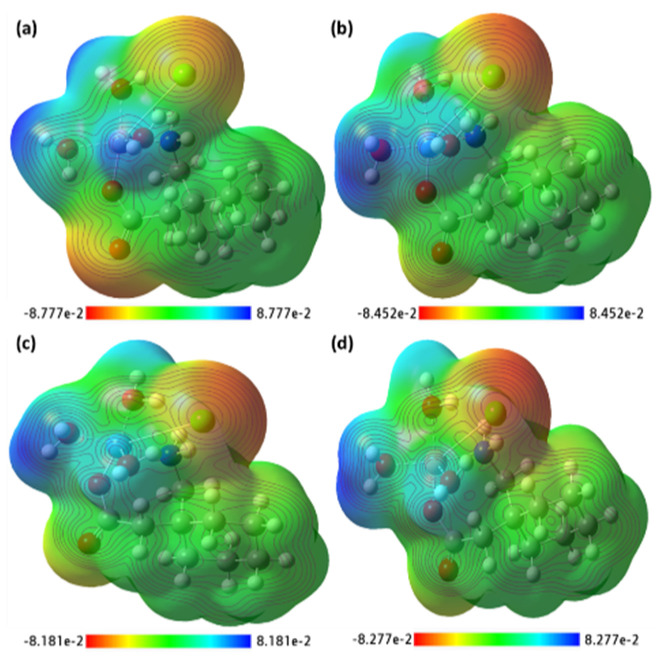
The MEP surface map of the synthesized metal complexes (**a**) [Mn(II)–(Gpn)], (**b**) [Co(II)–(Gpn)], (**c**) [Ni(II)–(Gpn)], and (**d**) [Cu(II)–(Gpn)] with the respective color scales.

**Figure 18:**
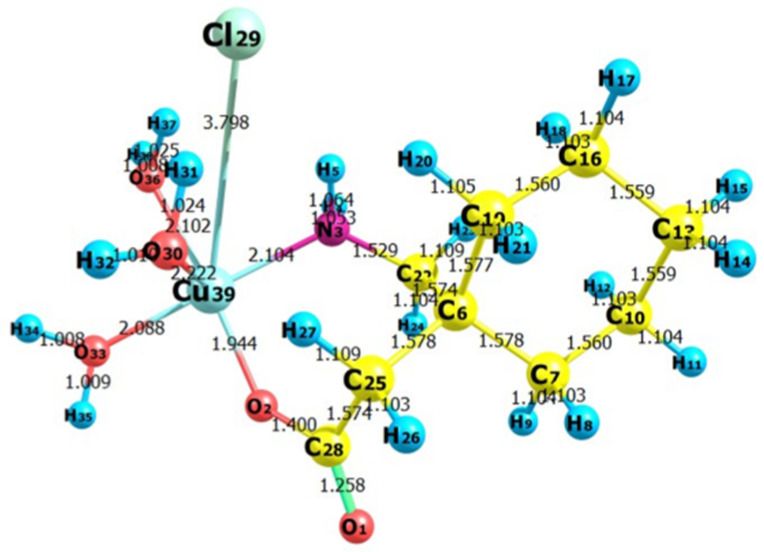
The optimized structure of the synthesized metal complex [Cu(II)–(Gpn)] showing bond lengths.

The strength of the electrostatic potentials for ([Mn(II)–(Gpn)], [Co(II)–(Gpn)], [Ni(II)–(Gpn)], and [Cu(II)–(Gpn)] was represented through the MEP map (Figure 17), in order to investigate the most electron rich and poor regions and to rationalize the noncovalent interactions. The electropositive regions are displayed in a blue color and the electronegative in a red color [49]. It was found that the area around the Cl and O atoms had strong negative electrostatic potential, and strong positive electrostatic potential could be seen around H_2_O, which shows the preferential binding sites over the molecule. The MEP surface map is represented in the color scale from deep red to deep blue [50]. From the optimized structure of [Cu(II)–(Gpn)], the bond lengths and bond angles were obtained (Figure 18, Table 6). The Mulliken charges for [Cu(II)–(Gpn)] were also calculated (Table 7). The shift in the Mulliken charges were observed in atoms of the synthesized metal complexes from the reactant moieties, which suggests the formation of different complexes. The result of the MEP is in agreement with the Mulliken charges.

**Figure 19:**
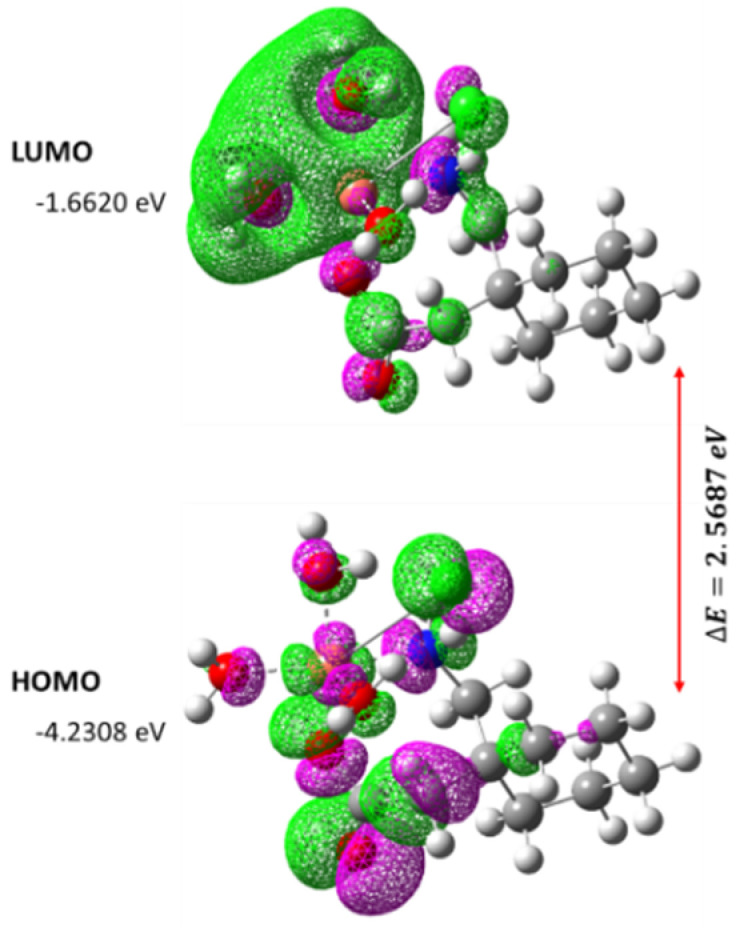
The spatial plot of HOMO and LUMO with their energy gap for the synthesized metal complex [Cu(II)–(Gpn)].

The IR frequencies were investigated through B3LYP/LanL2DZ (data not shown). The slight difference between the experimental and theoretical frequencies was due to the experimental values being obtained in the solid phase and theoretical values being acquired in the gas phase. The calculated vibrational frequencies varied to a smaller extent from the experimental results due to neglecting the incompleteness and anharmonicity of the basis set [51]. TD-DFT method was used to explore the nature of the electronic transitions in [Cu(II)–(Gpn)] in the gas phase. One broad electronic absorption band was obtained from TD-DFT at 569 nm. The HOMO (−4.2308 eV) to LUMO (−1.6620 eV) energy gap (∆E) was calculated to be 2.5687 eV. The spatial arrangements of the HOMO–LUMO, associated energies, and gap are represented in Figure 19 [52,53,54]. Some molecular parameters in the gas phase, based on HOMO–LUMO and optimized geometry, are presented in Table 8.

## 4. Conclusions

The gabapentin drug coordinated in a 1:1 ligand to metal ratio as a monobasic bidentate (ON) donor in all of the complexes. The analytical, magnetic, molar conductance, infrared vibrational motions, and electronic spectral study suggest that the structures shown in Figure 2 have an octahedral arrangement. The TEM morphology showed that the complexes were in nanosize ranges. The molecular docking results showed that the [Cu(II)–(Gpn)] metal complex interacted with both receptors more efficiently than the gabapentin drug and among all of them, [Cu(II)–(Gpn)]–serotonin (CuGS) had the highest binding energy value. A molecular dynamic (MD) simulation with a 100 ns run revealed that the CuGS complex possesses a more stable complex with the serotonin receptor than GpnS. The theoretical data obtained by DFT calculations agreed with the experimental data.

## Figures and Tables

**Figure 11:**
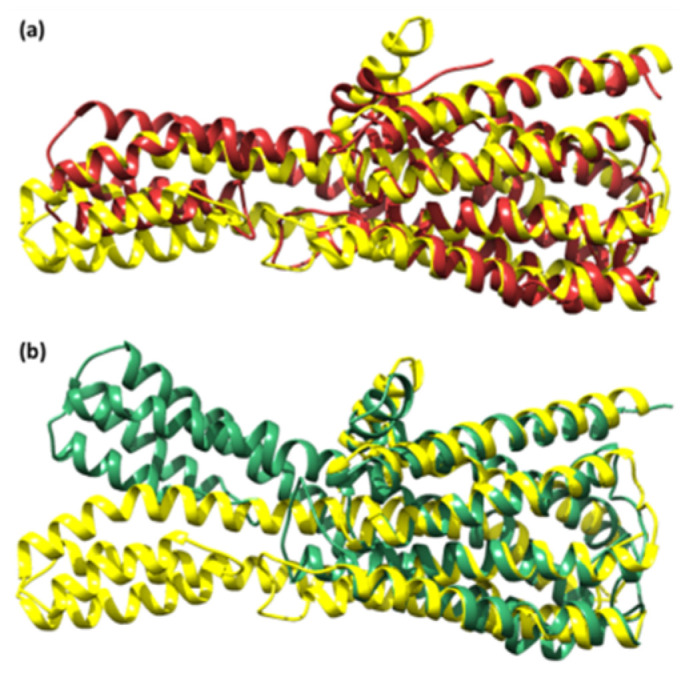
The superimposed structure of (**a**) the unbound serotonin receptor (yellow) and serotonin receptor after simulation (brown) for **GpnS** and (**b**) the unbound serotonin receptor (yellow) and serotonin receptor after simulation (green) for **CuGS**.

**Table 1:** Infrared spectral data (cm^−1^) of Gpn and its complexes.

Compounds	Frequencies, cm^−1^				
	ν_as_(COO)	ν_s_(COO)	δ(NH_2_)	ν(M-O)	ν(M-N)
Mn(II)	1650	1410	1568	617	502
Cu(II)	1660	1390	1558	612	516
Co(II)	1672	1398	1552	620	519
Ni(II)	1678	1395	1553	615	468

**Table 2:** The docking score of the four metal complexes docked with two receptors [serotonin (6BQH) and dopamine (6CM4)].

S. No.	Receptor Complex	Binding Free Energy (kcal/mol)	
		PDB: 6BQH	PDB: 6CM4
1	[Mn(II)–(Gpn)]	−6.9	−6.6
2	[Co(II)–(Gpn)]	−7.0	−6.7
3	[Ni(II)–(Gpn)]	−6.9	−6.4
4	[Cu(II)–(Gpn)]	−7.2	−6.5
5	Gpn	−5.1	−4.8

**Table 3:** The interactions of the [Cu(II)–(Gpn)] complex and Gpn with serotonin (6BQH).

S. No.	Receptor	Binding Free Energy (kcal/mol)	Interactions	
			H-Bond	Others
1	[Cu(II)–(Gpn)]	−7.2	Arg173, Thr109, His182, and Asn187	Asp172 (Attractive charge) and Ala176, Arg173 (Alkyl)
2	Gpn	−5.1	Thr109 and Ala108	Ala321 (Alkyl)

**Table 4:** The [Cu(II)–(Gpn)]–serotonin interaction results by Discovery Studio.

Name	Distance	Category
THR109:HG1–[Cu(II)–Gpn]:O	2.50	Hydrogen Bond
ARG173:HH11–[Cu(II)–Gpn]:O	2.63	Hydrogen Bond
ARG173:HH12–[Cu(II)–Gpn]:O	2.78	Hydrogen Bond
ASN187:HD22–[Cu(II)–Gpn]:O	2.43	Hydrogen Bond
[Cu(II)-Gpn]:H26–HIS182:O	2.59	Hydrogen Bond
[Cu(II)-Gpn]:H26–HIS182:O	2.49	Hydrogen Bond
[Cu(II)-Gpn]:H25–ASN187:OD1	2.35	Hydrogen Bond
[Cu(II)-Gpn]:H25–ASN187:OD1	2.50	Hydrogen Bond
ALA321–[Cu(II)–Gpn]	5.31	Hydrophobic

**Table 5:** The Gpn–serotonin interaction results by Discovery Studio.

Name	Distance	Category
Gpn:N–ASP172:OD2	5.34	Electrostatic
ALA108:HN–Gpn:O	2.30	Hydrogen Bond
THR109:HN–Gpn:O	1.85	Hydrogen Bond
THR109:HG1–Gpn:O	1.97	Hydrogen Bond
Cu(II)-Gpn:H–Gpn:O	2.91	Hydrogen Bond
ARG173–Gpn	4.93	Hydrophobic
ALA176–Gpn	5.04	Hydrophobic

**Table 6:** The bond lengths of the metal complex [Cu(II)–(Gpn)] obtained through DFT.

S. No.	[Cu(II)–(Gpn)] (B3LYP/LanL2DZ)			
	Atom No.	Bond Length (Å)	Atom No.	Bond Length (Å)
1	R(1–28)	1.258	R(16–17)	1.104
2	R(2–28)	1.400	R(16–18)	1.103
3	R(2–39)	1.944	R(16–19)	1.56
4	R(3–4)	1.053	R(19–20)	1.105
5	R(3–5)	1.064	R(19–21)	1.103
6	R(3–22)	1.529	R(22–23)	1.109
7	R(3–39)	2.104	R(22–24)	1.104
8	R(6–7)	1.578	R(25–26)	1.103
9	R(6–19)	1.577	R(25–27)	1.109
10	R(6–22)	1.574	R(25–28)	1.574
11	R(6–25)	1.578	R(30–31)	1.024
12	R(7–8)	1.103	R(30–32)	1.01
13	R(7–9)	1.104	R(30–39)	2.222
14	R(7–10)	1.560	R(33–34)	1.008
15	R(10–11)	1.104	R(33–35)	1.009
16	R(10–12)	1.103	R(33–39)	2.088
17	R(10–13)	1.559	R(36–37)	1.025
18	R(13–14)	1.104	R(36–38)	1.008
19	R(13–15)	1.104	R(36–39)	2.102
20	R(13–16)	1.559	R(29–39)	3.798

**Table 7:** The Mulliken atomic charges of the metal complex [Cu(II)–(Gpn)] atoms.

S. No.	Synthesized [Cu(II)-(Gpn)] Complex			
	Mulliken Atomic Numbers	Mulliken Atomic Charges	MullikenAtomic Numbers	Mulliken Atomic Charges
1	1O	−0.19391	21H	0.06922
2	2O	−0.11703	22C	−0.06068
3	3N	−0.34646	23H	0.08103
4	4H	0.20606	24H	0.09072
5	5H	0.23817	25C	−0.16982
6	6C	0.02926	26H	0.08199
7	7C	−0.13471	27H	0.11175
8	8H	0.07097	28C	0.21118
9	9H	0.07212	29Cl	−0.7276
10	10C	−0.13351	30O	−0.28504
11	11H	0.07166	31H	0.24647
12	12H	0.06446	32H	0.20389
13	13C	−0.13247	33O	−0.25834
14	14H	0.06717	34H	0.23457
15	15H	0.06992	35H	0.23367
16	16C	−0.1326	36O	−0.28583
17	17H	0.07643	37H	0.25414
18	18H	0.06574	38H	0.21341
19	19C	−0.13937	C39u	−0.03369
20	20H	0.08705		

**Table 8:** The various other theoretical molecular parameters of the metal complex [Cu(II)–(Gpn)].

Parameters	RB3LYP/LanL2DZ
Minimum SCF energy (a.u.)	−982.219370
Polarizability (a) (a.u.)	84.074494
Dipole moment (Debye)	7.662013
Zero point vibrational energy (kcal/mol)	241.62872
Total thermal energy (kcal/mol)	253.148
Electronic spatial extent (a.u.)	5378.5506
Frontier MO energies (eV)	
LUMO	−1.6620
HOMO	−4.2308
Gap (HOMO–LUMO)	2.5687

## Data Availability

All of the data supporting the reported results are available in the manuscript.

## Supplementary Materials

The following supporting information can be downloaded at: https://www.mdpi.com/article/10.3390/molecules27134311/s1, Figure S1: Receptor-ligand complex (a) CuGS and (b) GpnS in triclinic box solvated with water molecules and neutralized with 28 Na+ and 27 Cl- ions (0.15 M salt). Figure S2: Representation of (a) aromatic surface, (b) hydrophobic surface, (c) solvent accessible surface, (d) ionizability surface, and (e) Interpolated charge; between serotonin and metal complex [Cu(II)-(Gpn)].
