# Supplementary material for: Spectroscopic and Molecular Docking Studies of Cu(II), Ni(II), Co(II), and Mn(II) Complexes with Anticonvulsant Therapeutic Agent Gabapentin
Source: Molecules. 2022 Jul 5;27(13):4311. doi: 10.3390/molecules27134311 (PMC9268275; doi:10.3390/molecules27134311)
Supplement: Supplementary file 1 [file molecules-27-04311-s001.zip › molecules-1740396-supplementary.pdf]

Supplementary Data

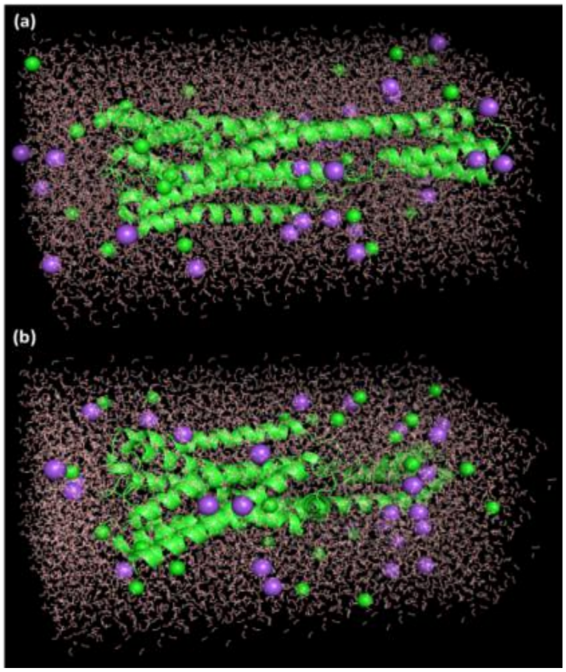

Figure S1. Receptor-ligand complex (a) CuGS and (b) GpnS in triclinic box solvated with water molecules and neutralized with 28 Na^{+} and 27 Cl^{-} ions (0.15 M salt).

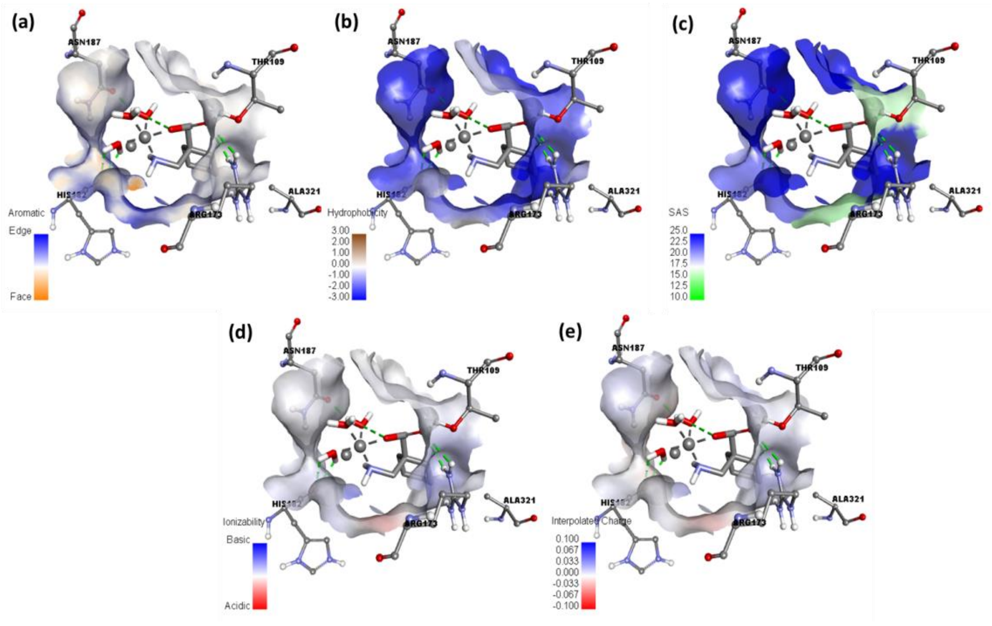

Figure S2. Representation of (a) aromatic surface, (b) hydrophobic surface, (c) solvent accessible surface, (d) ionizability surface, and (e) Interpolated charge; between serotonin and metal complex [Cu(II)-(Gpn)].
